# Intraoperative Augmented Reality for Complex Glioma Resection: A Case Report

**DOI:** 10.7759/cureus.57717

**Published:** 2024-04-06

**Authors:** Rachel Hunt, Lisa Scarpace, Jack P Rock

**Affiliations:** 1 Neurosurgery, Henry Ford Health, Detroit, USA; 2 Neurosurgery, Henry Ford Health, Pittsburgh, USA

**Keywords:** technology, image guidance, neuronavigation, mixed reality, glioma, augmented reality

## Abstract

Augmented reality (AR) is an emerging technology that can display three-dimensional patient anatomy in the surgeons’ field of view. The use of this technology has grown considerably for both presurgical and intraoperative guidance. A patient diagnosed with breast cancer started to experience numbness in the left hand, which progressed to weakness in the left hand and arm. An MRI was performed demonstrating a 2.9 cm X 1.8 cm lesion with extensive surrounding edema in the posterior fronto-parietal lobes. Surgery was recommended for presumed metastatic disease. Preoperatively, an AR system and Brainlab navigation were registered to the patient. AR, traditional navigation, and ultrasound were all used to localize the lesion and determine the craniotomy site and size. The tumor was removed along the direction of the lesion. Intraoperatively, we used AR to reexamine the tumor details and could appreciate that we had to redirect our surgical trajectory anteriorly and laterally in order to follow along the main axis of the tumor. In doing this, we were able to more confidently remain with the tumor, which by this time was poorly defined by 2D navigation and by direct vision. Postoperative MRI confirmed gross total removal of the tumor. The patient had an uneventful postoperative course with resolution of preoperative symptoms and the final surgical pathology was grade 4 glioblastoma. Here, we describe the valuable use of AR for the resection of a glioma. The system has a seamless registration process and provides the surgeon with a unique view of 3D anatomy overlaid onto the patient’s head. This exciting technology can add tremendous value to complex cranial surgeries.

## Introduction

In the field of neurosurgery, where precision is paramount, the advent of new imaging technologies and neuronavigation has heralded a transformative era in surgical planning and execution [1,2]. Despite advancements, there remain gaps in surgeons’ ability to navigate and appreciate the three-dimensional organization of patient neuroanatomy. In particular, the process of mentally converting 2D medical imaging into a 3D structure can be particularly difficult, especially when dealing with lesions of irregular shapes and sizes.

Augmented reality represents a unique opportunity for the surgeon to study and visualize patient anatomy in 3D. This technology superimposes 3D digital models onto a surgeon’s real-world field of view [3-6]. Here, we describe a complex glioma resection whereby augmented reality (AR) was used to better understand and approach the lesion both preoperatively and intraoperatively. In this report, the AR system used allowed the surgeon to display the 3D model overlaid onto the patient’s head, such that the surgeon could appreciate the location and orientation of the full lesion as the patient was in the operative position. With this case, we highlight particularly valuable features of augmented reality in hopes that it drives greater adoption and integration of this technology into neurosurgical care.

## Case presentation

**Figure 1:**
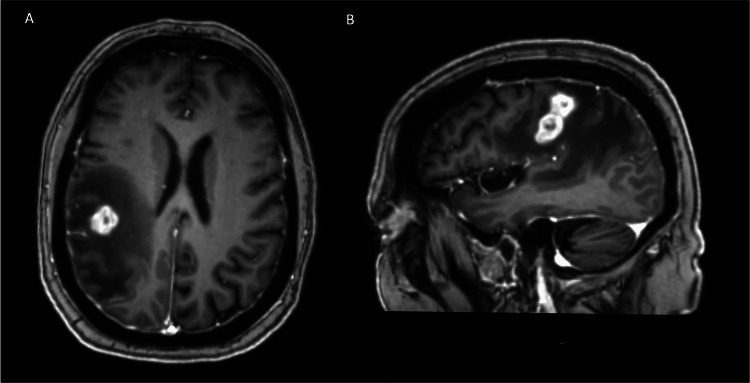
Preoperative imaging A) Axial and B) sagittal view of patient preoperative MRI

A 69-year-old female patient was diagnosed with breast cancer, which was followed by surgery and radiotherapy. The patient started to experience numbness in the left hand three weeks before surgery, which progressed to weakness in the left hand and arm. An MRI was performed, demonstrating a 2.9 cm X 1.8 cm lesion with extensive surrounding edema. The lesion was located in the posterior frontoparietal lobes posterior to the motor cortex. Surgery was recommended for presumed metastatic disease (Figure 1). Prior to surgery, a CT of the chest, abdomen, and pelvis was negative.

Augmented reality technology platform

An AR system was used both preoperatively and intraoperatively. The software (Hoth Intelligence, Philadelphia, Pennsylvania, USA) operates on the Microsoft Hololens 2 HMD (Microsoft Corporation, Redmond, Washington, USA). It is an untethered optical see-through head-mounted unit that allows users to visualize digital content (i.e., holograms, images, screens) in their real-world field of view [7-9].

Mixed reality presurgical planning

**Figure 2:**
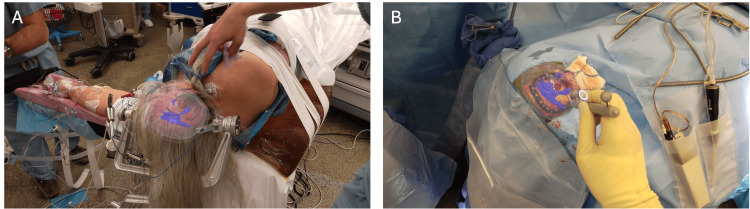
Surgeon's augmented reality view through the headset User’s view through the augmented reality (AR) headset displaying a 3D model overlaid onto the patient's head a) preoperatively and b) intraoperatively. The 3D model consists of the face (gray), skull (clear), tumor (red), and ventricles (blue).

The AR technology used for this case operates out of a small wireless headset -- the Microsoft Hololens 2 (Microsoft Corporation). The system overlays/registers 3D models of the patient’s anatomy onto the head via a markerless registration process. While wearing the headset, the surgeon looks at the patient’s face, at which point the headset sensors capture facial and surface information from the patient. Data captured through the headset is aligned with the MRI image and the 3D model is registered and overlaid on the patient’s head. The system operates entirely out of the headset and does not require external cameras, monitors, or computer systems. The 3D model for this case consisted of multiple layers including face (gray), ventricles (blue), tumor (red), and skull (white/clear). The patient's 3D model was registered onto the patient’s head in the operating room (Figure 2A). Once registered onto the head, the surgeons were able to move the head and visualize the underlying lesion in 3D. After draping the 3D model remains overlaid onto the patient’s head, allowing the surgeon to have AR visualization throughout the case (Figure 2B).

Operative and postoperative course

**Figure 3:**
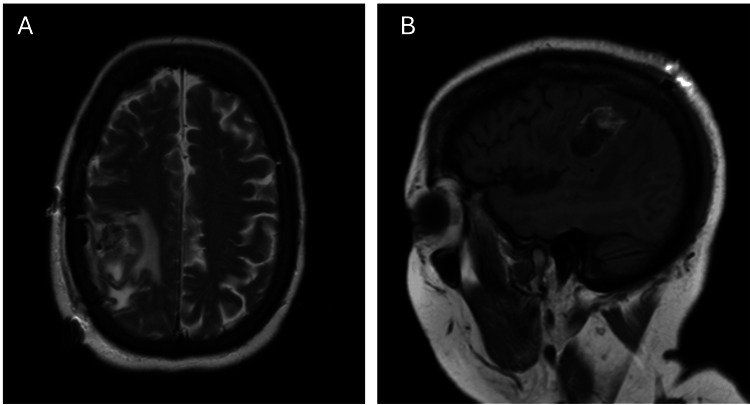
Postoperative Imaging A) Axial and B) Sagittal view of patient postoperative MRI. Gross total removal of the tumor was confirmed.

After anesthesia and antibiotics, the patient was placed supine with bolsters under the hip and shoulder. The patient was then placed in the head pins and both the AR system and Brainlab navigation were registered. AR, traditional navigation, and ultrasound were used to localize the lesion and determine the craniotomy site and size. After prepping and draping, the incision was made, the bone plate was removed, and the dura opened. The brain was full in the location of the tumor and a cortisectomy led directly to the tumor. At first, the lesion seemed encapsulated but gradually the capsule thinned and the capsule was no longer apparent. We removed the tumor along the direction of the lesion until we came to the normal-appearing brain on the medial aspect of the lesion. The lesion was identified posterior to the motor cortex however cortical and subcortical mapping were performed to confirm that the resection did not encroach upon motor fibers. The intraoperative pathology specimens came back with a high-grade glioma. We reconfirmed our surgical directions with ultrasound but this was, at best, confusing. So, we used AR to re-examine the tumor details and could appreciate that we had to redirect our surgical trajectory anteriorly and lateral to follow along the main axis of the tumor. In doing this, we were able to more confidently remain with the tumor, which by this time was poorly defined by 2D navigation and by direct vision. When we came to more normal-appearing white matter around the area of the tumor, we stopped the resection, confirmed by ultrasound the extent of resection, and, routinely closed the craniotomy. Intraoperative motor monitoring verified that we had not intruded upon descending motor fibers. Postoperative MRI confirmed the gross total removal of the tumor (Figure 3). The patient had an uneventful postoperative course with resolution of preoperative symptoms and the final surgical pathology was grade 4 glioblastoma.

## Discussion

The field of neurosurgery has continued to integrate augmented reality into clinical care and identify new use cases and applications of this technology to improve patient safety [10-14]. As the technology becomes more accurate and user-friendly, the role and value of augmented reality in procedures continue to grow. Here, we present a case where the addition of augmented reality was used preoperatively and intraoperatively for visualizing the three-dimensional shape and direction of a glioma lesion.

In cases such as the one presented here, even the most experienced neurosurgeon can struggle to appreciate the three-dimensional shape of the tumor based on 2D imaging and 2D neuronavigation. This case presented a particular challenge given the irregular shape and path the tumor took through the brain. Traditional navigation and ultrasound were used; however, the 2D visualization afforded by these systems was limiting and made proper understanding and visualization of the lesion a challenge. The addition of augmented reality confirmed and facilitated a better understanding of the tumor’s dimensions. In this particular case, the ability to directly overlay the 3D model onto the patient's head was invaluable for both planning the approach and for confirming that once within the tumor, the surgeon was following the correct trajectory along the tumor in order to safely and completely remove the lesion.

In cranial neurosurgery, the use of augmented reality has predominantly been limited to preoperative visualization [15-17]. In particular, the technology is traditionally used prior to the start of a case in order to visualize complex anatomy in 3D. A few cases use augmented reality intraoperatively either using a head-mounted display or by combining AR visualization with a microscope display [18-22]. In this case, a head-mounted display was used both preoperatively and intraoperatively in synergy with traditional neuronavigation to navigate and resect a difficult tumor. This is a prime example of the value of augmented reality, as the ability to visualize the patient's anatomy in three dimensions overlaid onto the head was critical to the case. Additionally, a major advantage of the system used for this case is the registration process. Registration is fast and importantly does not require fiducials for the system to register the 3D model with the patient. Lastly, the system operates solely out of a small headset and thus requires little to no space in the operating room. Altogether, the fast registration process and small size make it an excellent adjunct to neuronavigation.

There are many cases similar to the one presented here where traditional 2D navigation and the required mental conversion to 3D are challenging, especially for junior neurosurgeons [23]. While this demonstrates an optimal use of augmented reality, further work is required if this technology is to be readily adopted in the field. For example, in this report, we did not quantify the correspondence between the neuronavigation display and the augmented reality display. Visually, the 3D AR model appeared in the correct location and overlaid directly on the 2D neuro navigation displays. However, future work that measures the relative accuracy between the AR system and traditional navigation is warranted. Additionally, there is certainly potential for this technology to be used as a traditional neuronavigation system, and thus preclinical and clinical studies addressing the efficacy of AR as a neuronavigation system will be required.

## Conclusions

Augmented reality facilitates a better understanding of patient anatomy by allowing providers to evaluate anatomical structures in 3D. In their report, we describe the valuable use of augmented reality for the resection of a glioma. The system has a seamless registration process and provides the surgeon with a unique view of 3D anatomy overlaid onto the patient’s head. The AR system provided new information about the patient's anatomy that was not easily appreciated using traditional image guidance. As the use of augmented reality continues to expand, this technology will add tremendous value to complex cranial procedures.
